# Modulation of Gene Expression in Liver of Hibernating Asiatic Toads (*Bufo gargarizans*)

**DOI:** 10.3390/ijms19082363

**Published:** 2018-08-10

**Authors:** Long Jin, Jian Ping Yu, Zai Jun Yang, Juha Merilä, Wen Bo Liao

**Affiliations:** 1 Key Laboratory of Southwest China Wildlife Resources Conservation (Ministry of Education), China West Normal University, Nanchong 637009, China; longjin07@126.com (L.J.); jianpingyu@163.com (J.P.Y.); zaijunyang@cwnu.edu.cn (Z.J.Y.); 2 Key Laboratory of Artificial Propagation and Utilization in Anurans of Nanchong City, China West Normal University, Nanchong 637009, China; 3 Institute of Eco-Adaptation in Amphibians and Reptiles, China West Normal University, Nanchong 637009, China; 4 Ecological Genetics Research Unit, Department of Biosciences, University of Helsinki, P.O. Box 65, FI-00014, 00100 Helsinki, Finland; juha.merila@helsinki.fi

**Keywords:** *Bufo gargarizans*, gene expression, hibernation, liver, energy conservation

## Abstract

Hibernation is an effective energy conservation strategy that has been widely adopted by animals to cope with unpredictable environmental conditions. The liver, in particular, plays an important role in adaptive metabolic adjustment during hibernation. Mammalian studies have revealed that many genes involved in metabolism are differentially expressed during the hibernation period. However, the differentiation in global gene expression between active and torpid states in amphibians remains largely unknown. We analyzed gene expression in the liver of active and torpid Asiatic toads (*Bufo gargarizans*) using RNA-sequencing. In addition, we evaluated the differential expression of genes between females and males. A total of 1399 genes were identified as differentially expressed between active and torpid females. Of these, the expressions of 395 genes were significantly elevated in torpid females and involved genes responding to stresses, as well as contractile proteins. The expression of 1004 genes were significantly down-regulated in torpid females, most which were involved in metabolic depression and shifts in the energy utilization. Of the 715 differentially expressed genes between active and torpid males, 337 were up-regulated and 378 down-regulated. A total of 695 genes were differentially expressed between active females and males, of which 655 genes were significantly down-regulated in males. Similarly, 374 differentially expressed genes were identified between torpid females and males, with the expression of 252 genes (mostly contractile proteins) being significantly down-regulated in males. Our findings suggest that expression of many genes in the liver of *B. gargarizans* are down-regulated during hibernation. Furthermore, there are marked sex differences in the levels of gene expression, with females showing elevated levels of gene expression as compared to males, as well as more marked down-regulation of gene-expression in torpid males than females.

## 1. Introduction

Hibernation is an effective energy conservation strategy adopted by endotherms to cope with the adverse environmental conditions during the winter [1,2,3]. During the hibernating period, the metabolic rate, heart beat rate, and oxygen consumption of hibernating small mammals can experience remarkable reductions [4,5,6]. Estivation is a survival strategy for some poikilotherms (e.g., amphibians and reptiles) against the dry season in summer [7,8]. In amphibians, the metabolism during torpor or estivation may be depressed by as much as 80% of the normal metabolic rate [8,9]. Marked physiological transitions in energy utilization take place in torpid amphibians [1].

For instance, lipids stored in white adipose tissue can be hydrolysed to free fatty acids and glycerol with the enzyme lipase, which will last both the hibernating period and the active period, but conversion to ketone bodies and glucose occurs in liver during the hibernating period [10]. The ketone bodies are an important source of energy, and can be transmitted to other tissues [11]. Hence, it is thought that the liver plays a major role in physiological regulation of metabolism during the hibernating period [1,12,13].

Until now, the molecular and genetic basis of hibernation physiology has been extensively investigated in mammals using large-scale genomic approaches [14,15,16]. Available evidence suggests that many genes involved in metabolism are differentially expressed during the hibernating period. For instance, most genes involved in carbohydrate, lipid, and amino acid metabolism, detoxification and molecular transport in liver tissue are down-regulated in hibernating squirrels [17,18]. A total of 1358 differentially expressed genes in the liver between active and hibernating bats were mainly involved in metabolic depression, shifts in utilization of different energy sources, immune function, and stress responses [12]. Similarly, genes playing roles in protein biosynthesis and fatty acid catabolism with coordinated reduction of transcription of genes playing roles in lipid biosynthesis and carbohydrate catabolism are up-regulated in the liver of *Ursus americanus* [13]. Although these studies have advanced our understandings of liver physiology in mammals, what happens for gene expression in the liver of hibernating anurans remains unexplored.

The Asiatic toad (*Bufo gargarizans*) is widely distributed in China, occurring at elevations from 120 to 1500 m [19]. The species is an explosive breeder with a relatively short spawning period (6–24 days) [20], with typical breeding habitats concentrated along the vegetated edges of large, still water bodies. Competition for mating is fierce among males [19,21], and the species experiences a period of hibernation from early-December to mid-February and the mating behaviour occurs as soon as the hibernation period ends. Hence, *B. gargarizans* is a suitable model for investigating the physiological and molecular mechanisms underlying metabolic suppression during the hibernating period. However, no information on gene expression differences among active and hibernating individuals is currently available.

The next-generation sequencing (NGS) technologies have provided tools to conduct genome-wide studies of non-model organisms with limited genomic resources [14,15,16,17,22]. Amongst other things, they have opened the door to obtain cDNA fragments from transcriptomes with reasonably complete coverage at a low cost [23]. The aim of this study is to compare liver tissue transcriptomes of active and torpid toads with the aid of NGS technology and, in particular, to identify differentially expressed genes that are contributing to the hibernation phenotype. A secondary goal is to compare sex differences in gene expression.

## 2. Results

### 2.1. Transcriptome Sequencing, Read Assembly, and Mapping

Transcriptome sequencing produced 20,836,817, 25,678,062, 21,610,351 and 21,463,798 reads from active female, active male, torpid female, and torpid male libraries, respectively (Table 1). The corresponding numbers of total bases generated were 5,240,784,114, 6,460,736,308, 5,426,895,884 and 5,393,246,014 bp, respectively (Table 1). After de novo assembly with Trinity, 27,349 unigenes (length > 500 bp) were recovered, the mean length of these was 849.04 bp. For further analyses of differential gene expression in four library types, the raw reads from the libraries were separately mapped to the assembled contigs (length > 500 bp) that functioned as a transcriptome reference database. Table 1 provides information about the reads mapped to the four libraries, and the mapping rate was 78.22%–80.28%. The distribution of contigs to different length intervals is shown in Figure S1.

### 2.2. Identification and Validation of Differentially Expressed Genes (DEGs)

**Figure 1:**
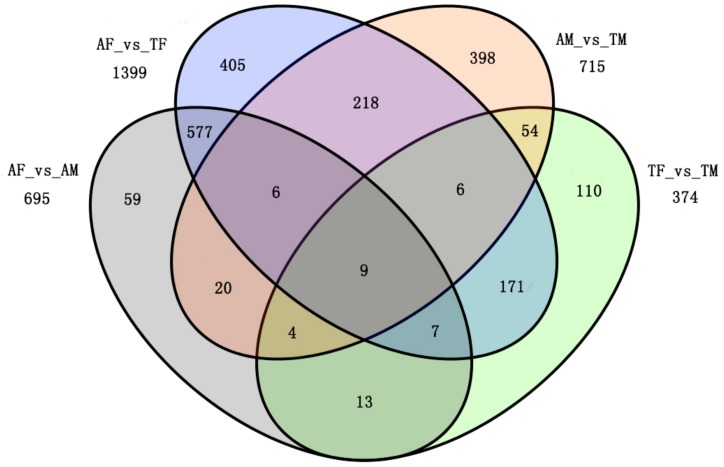
Venn diagram showing differentially expressed genes among active females (AF), active males (AM), torpid females (TF), and torpid males (TM) for transcripts with log_2_ fold change ≥2. (AF-vs.-TF: 1399 differentially expressed genes; AM-vs.-TM: 715 differentially expressed genes; AF-vs.-AM: 695differentially expressed genes; TF-vs.-TM: 374 differentially expressed genes).

Applying a filter of false discovery rate (FDR) ≤ 0.01 and the absolute value of log_2_ fold change ≥2, we identified 695 differentially expressed genes between active females and males (AF-vs.-AM), of which 655 genes were down-regulated and 40 genes were up-regulated in the active males (Figure 1 and Table S1).

A total of 1399 differentially expressed genes were identified between active and torpid females (AF-vs.-TF), of which 1004 genes were down-regulated and 395 genes were up-regulated in the torpid females (Figure 1 and Table S1). By comparing 715 differentially expressed genes between active and torpid males (AM-vs.-TM), we found that 378 genes were down-regulated and 337 genes were up-regulated in the torpid males (Figure 1 and Table S1). A total of 374 differentially expressed genes were identified between torpid females and males (TF-vs.-TM) of which 252 genes were down-regulated and 122 genes were up-regulated in the torpid males (Figure 1 and Table S1). Considering that genes highly expressed in liver may play important roles in the physiological functions, the genes with the top 10 reads per kilobase per million mapped reads (RPKM) that were differentially expressed in the four groups are listed in Table 2, Table 3, Table 4 and Table 5.

Analysing the differentially expressed genes between active and torpid toads (AF-vs.-TF and AM-vs.-TM), we find that most genes were significantly down-regulated in torpid livers, and the gene with the maximum log_2_-fold change value (up to −6.44 and −4.13, respectively) was c44660.graph_c0. Moreover, two genes, one involved in fatty acid metabolism (c111752.graph_c0) and another facilitating glucose transportation (c90064.graph_c0), were down-regulated in torpid livers. By contrast, the gene (c122333.graph_c0) encoding a heat shock protein was up-regulated during torpor. The common genes that were differentially expressed in both sexes during the hibernating period were shown in Table S2. By comparing differentially expressed genes between active and torpid females (AF-vs.-TF), the nicotinamide adenine dinucleotide phosphate (NADPH)-cytochrome P450 reductase isoform X1 (c120235.graph_c0) was up-regulated during the hibernating period. Likewise, the genes encoding contractile proteins (c87275.graph_c0, c115995.graph_c0 and c122569.graph_c1) were up-regulated in torpid liver.

When comparing differentially expressed genes between females and males in active period (AF-vs.-AM), the down-regulated genes included NADPH-cytochrome P450 reductase isoform X1 (c120235.graph_c0), the gene encoding several key rate-limited regulated enzymes of glycolysis (c114360.graph_c0, c123807.graph_c0, c119772.graph_c0, c123584.graph_c0, c117125.graph_c0) and amino acid transport and metabolism (c104930.graph_c0, c107734.graph_c0, c118687.graph_c0, c118905.graph_c0, c119432.graph_c0, c84432.graph_c0) in active males. When comparing differentially expressed genes between torpid females and torpid males (TF-vs.-TM), the gene encoding contractile proteins (c87275.graph_c0, c115995.graph_c0, and c122569.graph_c1) were down-regulated in torpid males.

**Figure 2:**
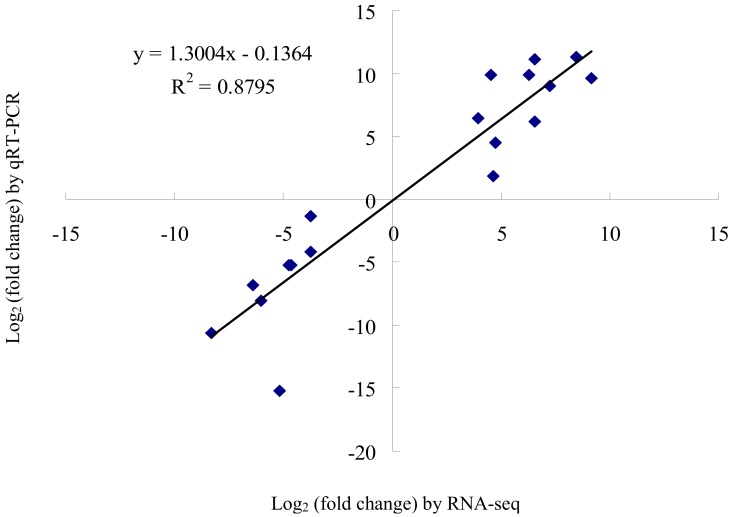
Comparison of expression levels measured by RNA-Seq and qRT-PCR for 18 selected transcripts. The two independent measures of gene expression displays a positive correlation.

To test the validity of our measurements, we analysed the RNA sequence data of 18 randomly-selected genes with the results from qRT-PCR experiments to detect the relative mRNA expression changes of the selected genes in the four groups (AF-vs.-AM, AF-vs.-TF, AM-vs.-TM, and TF-vs.-TM). The positively and highly significant correlation coefficient (*R*^2^ = 0.8795) indicated that the two independent measures of gene expression exhibited similar patterns, testifying the reliability of the RNA-Seq data (Figure 2).

### 2.3. Sequence Annotation

BlastX searches were made in various protein databases, and the GenBank NR, Swiss-Prot, PFAM, KEGG, COG, GO, and KOG databases were employed for annotation of the 21,261 unigene sequences (Table 6). Significant matches were found for 5760 (27.09%) unigenes in the COG database, 10,554 (49.64%) in the GO database, and 10,798 (50.78%) in the KEGG database. In the KOG, PFAM, Swiss-prot, and NR databases, 13,424 (63.13%), 15,330 (72.10%), 10,902 (51.27%), and 20,046 (94.28%) significantly matched unigenes were obtained, respectively.

In the GO annotation, the 10,554 unigenes were allocated to one or more GO terms on the basis of sequence similarity (Table 6). The three main categories of GO annotations were for cellular components (25,872 genes; 36.61%), for molecular function (12,806; 18.12%), and for biological processes (31,985; 45.27%). For cellular components, genes involved in “cell” and “cell part” terms were the most common (Figure S2a–d). In the category of molecular function, the term “binding” was in the highest proportion of annotations, followed by “catalytic activity” (Figure S2a–d). For biological processes, the most frequent GO term was “cellular process”, followed by “single-organism process” and “metabolic process” (Figure S2a–d). The GO analysis revealed a significant enrichment of genes associated with response to stress in the biological processes category: these genes were up-regulated in both sexes during the hibernating period (Table 7).

**Figure 3:**
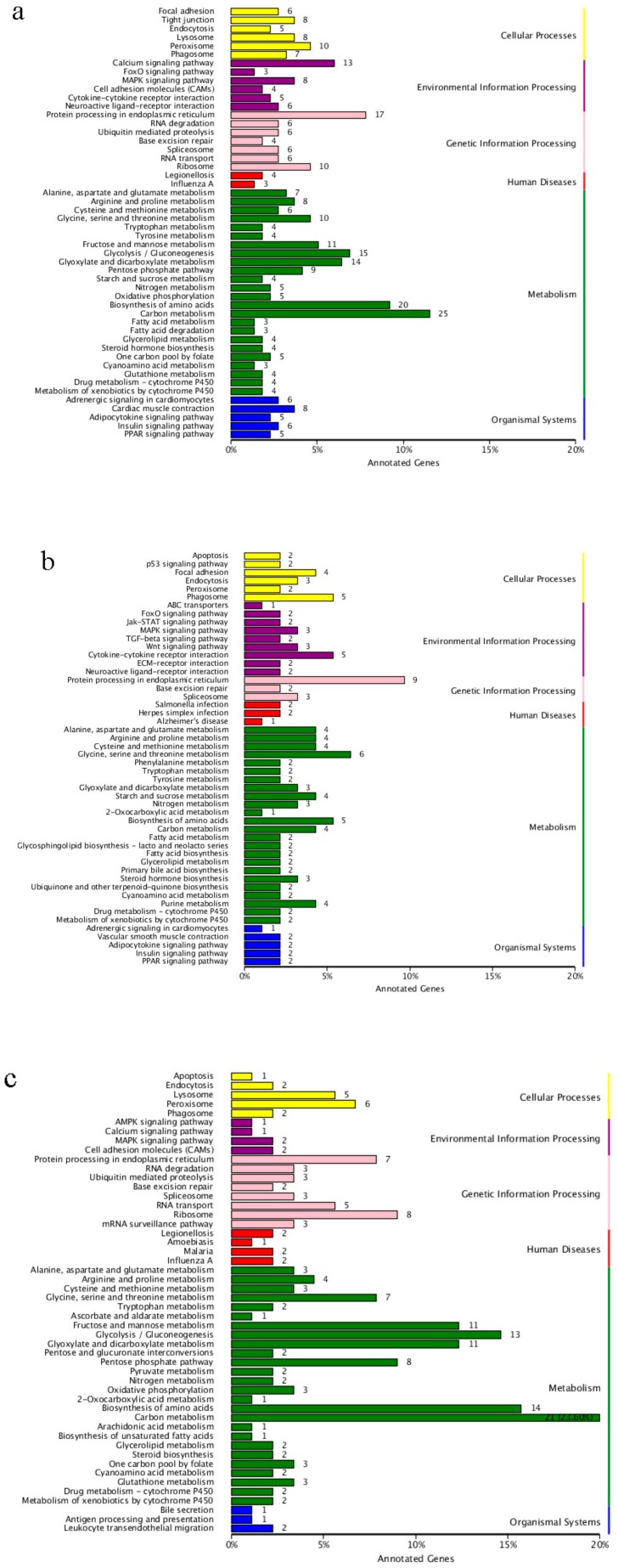

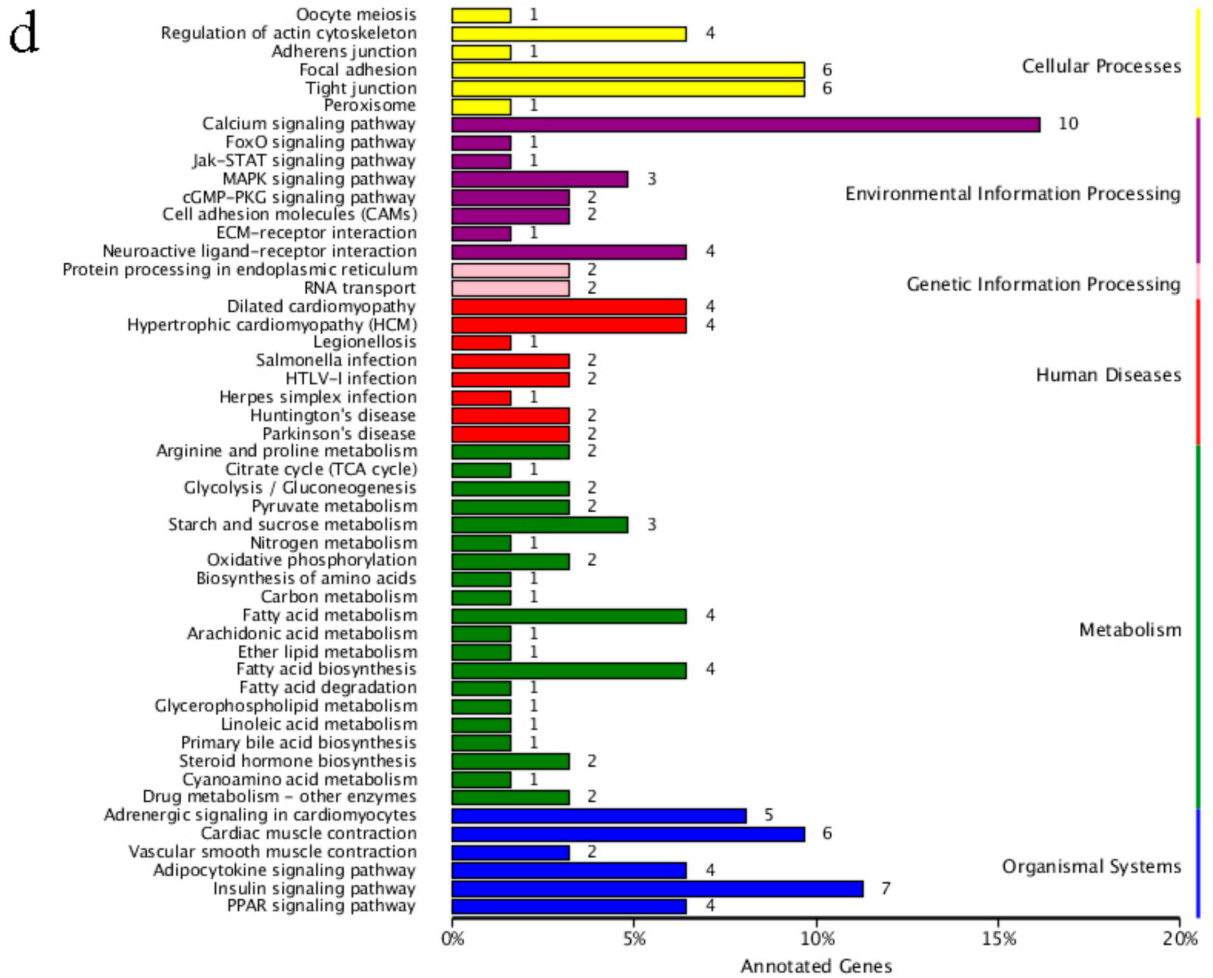
KEGG pathway enrichment analysis of differentially expressed genes in four comparisons of samples, active female vs. torpid female (**a**), active male vs. torpid male (**b**), active female vs. active male (**c**), and torpid female vs. torpid male (**d**).

To further explore the biological pathways, the unigene sequences were mapped with the KEGG Pathway Tools. These were classified into six categories: cellular process, environmental information processing, genetic information processing, human diseases, metabolism and organismal systems. This process for active and torpid states assigned 380 unigenes to a total of 143 pathways in females and 157 unigenes to a total of 98 pathways in males, respectively. For metabolism, the genes in the carbon metabolism (ko01200) and biosynthesis of amino acids (ko01230) involved in “carbohydrate transport and metabolism” and “amino acid transport and metabolism” were down-regulated during torpor in both sexes (Figure 3a,b). Meanwhile, genes including the glycolysis/gluconeogenesis (ko00010), glyoxylate and dicarboxylate metabolism (ko00630), fructose and mannose metabolism (ko00051), pentose phosphate pathway (ko00030), and fatty acid metabolism (ko01212) involved in “carbohydrate transport and metabolism” and “lipid transport and metabolism” were down-regulated in the torpid females (Figure 3a). In addition, genes in the calcium signalling pathway (ko04020) and cardiac muscle contraction (ko04260) involved in “calcium ion transport” and “muscle contraction” were up-regulated in the torpid females in environmental information processing and organismal systems categories (Figure 3a).

This process for females and males assigned 149 unigenes to a total of 86 pathways in the active state and 136 unigenes to a total of 60 pathways in the torpid state, respectively (Figure 3c,d). For metabolism, the genes involved in the carbon metabolism (ko01200), glycolysis/gluconeogenesis (ko00010), fructose and mannose metabolism (ko00051), pentose phosphate pathway (ko00030) and biosynthesis of amino acids (ko01230) were down-regulated in active males (Figure 3c). For environmental information processing and organismal systems, the genes including the calcium signalling pathway (ko04020) and cardiac muscle contraction (ko04260) involved in “calcium ion transport” and “muscle contraction” were down-regulated in the torpid males (Figure 3d).

### 2.4. Body Mass and Fat-Body Mass

Body mass of the torpid toads was significantly lower than that of active ones (two-way ANOVA: *F*_1,36_ = 5.069, *p* = 0.031; Tables S3 and S4). Fat-body mass in the torpid toads was significantly lower than in active ones (two-way ANOVA: *F*_1,36_ = 5.051, *p* = 0.031; Tables S3 and S4).

## 3. Discussion

### 3.1. Metabolic Enzymes

As an important strategy for energy conservation, reduced metabolic rate plays a critical role for survival in harsh winter in many mammals [4]. The minimum metabolic rate during torpor can be as low as 4.3% of the basal metabolic rate [6], enabling hibernators to save 90% of normal energy usage [24]. How do the hibernating animals mediate a low level of their metabolic rate? Changed traits and levels of gene expression permit them to display a diversity of phenotypes even with a common genotype [1]. The variations of physiology in an organism shifting between active and torpid states may result from the changed expression of genes encoding proteins where that specific functions can be served in physiologcal process [12]. We found that most of the genes involved in metabolic pathways were down-regulated during torpor. For example, the genes were down-regulated and were likely to play a crucial role in energy metabolism by encoding an important enzyme (NADPH-cytochrome P450 reductase isoform X1). This enzyme takes part in an extra-hepatic P450 electron transport pathway and contributes to the accumulation of hepatic lipids [25,26]. The above-mentioned example suggests that transcriptional changes resulted in metabolic adjustment between active and torpor states. Similarly, the results from torpid *Rhinolophus ferrumequinum* reveal co-down-regulation of genes involved in the glycolytic pathway playing a central role in metabolic suppression during torpor [12,14].

Amphibians, like other animals, undergo critical shifts in their energy utilization pathways during torpor, specifically switching from carbohydrate to fat-based metabolism [1]. For *B. gargarizans*, the fat mass is highest in July and lowest in March, and lipids are the primary energy source during the hibernating period [27], as in most torpid animals [28]. In our material, fat-body mass in torpid toads was significantly lower than in active ones (Tables S3 and S4), suggesting active utilization of fat-bodies during the hibernating period.

By comparing the gene expression between active and torpid females (AF-vs.-TF), we found that genes encoding several key rate-limited regulated enzymes of glycolysis, lipid transport and metabolism and protein synthesis (c21604.graph_c0, c85444.graph_c0, and c109713.graph_c0) were expressed at lower levels during torpor. Likewise, the five genes (c116341.graph_c0, c113894.graph_c0, c115957.graph_c0, c90064.graph_c0, and c115215.graph_c0) facilitating glucose transportation were down-expressed in torpid females. Similarly, we also found that genes encoding facilitated glucose transporter and amino acid metabolic processes were down-expressed in torpid males when compared to active males (AM-vs.-TM). These findings align with the results of Xiao et al. [12] who find that some differentially expressed genes are associated with the glycolytic pathway and lipid metabolism in the liver between active and torpid *R. ferrumequinum*.

NADPH-cytochrome P450 reductase isoform X1 (c120235.graph_c0) and several genes encoding several key rate-limited regulated enzymes of glycolysis (c114360.graph_c0, c119772.graph_c0, c117125.graph_c0) were down-regulated in active males as compared to active females (AF-vs.-AM). As the basal metabolic rate (BMR) increases with body mass and body surface area [29,30], and females are the larger sex in *B. gargarizans*, we would expect females to exhibit higher BMR than males. Moreover, the BMR in females is expected to be elevated by pregnancy [31,32]. In fact, torpid females are carrying developing eggs during the hibernating period, thus, in order to guarantee the normal development of their eggs, the females are not allowed to decrease their metabolism to the level which is similar to that of males.

### 3.2. Contractile Proteins

It has been shown that titin, serving as a molecular spring, results in elasticity and passive muscle stiffness [33] which is likely to play a critical role in mediating heart function at extremely low temperatures. Hence, titin is an important determinant in diastolic filling of heart. There is evidence that, for *Citellus undulates*, the 3400-kDa isoform of titin is expressed predominantly in both skeletal and cardiac muscle during the hibernating period [34]. In this study, the genes encoding contractile proteins (c87275.graph_c0, c115995.graph_c0, c122569.graph_c1) were up-regulated in torpid females as compared to active females (AF-vs.-TF). In line with our findings, the expression of genes in the heart altering contractility and Ca^2+^ handling are higher in the hibernating season than in the active season by the thirteen-lined ground squirrel (*Spermophilus tridecemlineatus*) [35].

The genes encoding contractile proteins (c87275.graph_c0, c115995.graph_c0, and c122569.graph_c1) were down-expressed in torpid males as compared torpid females (TF-vs.-TM). Blackburn and Calloway suggest that oxygen uptake increases after exercise for pregnant woman [31]. In contrast to males, females continue their reproductive investment during the hibernating period by developing eggs. This suggests that torpid females may be forced to increase their oxygen uptake during the hibernating period more than torpid males and, hence, also up-regulation of genes involved in the production of contractile proteins.

### 3.3. Anti-Stress Response

It has been found by several studies that, during the hibernating period, protein synthesis and proteolysis are often in a state of being suppressed [4,36]. The elevated expression of multi-family heat shock protein (*HSP*) genes in this study suggests that the preservation of the proteome from protein folding during torpor is a function that is preserved even during torpor. Stresses, such as hypoxia or ischemia, induce protein mis-folding during torpor, which also occurs in the pathological conditions related to neurodegenerative diseases and brain injury [37,38,39]. In the current study, we found many *HSP* genes were up-regulated during torpor (cf. comparisons: AF-vs.-TF and AM-vs.-TM), suggesting that elevated expression of *HSP* may play a specific role in protein homeostasis to maintain tissue-specific functions. This inference is supported by the fact that the up-regulation of *HSP* genes have also been reported in studies of hibernating mammals [40,41,42].

## 4. Experimental Section

### 4.1. Animals and Sample Preparation

The specimens used in this study were collected with permission (# 17001) from the Ethical Committee for Animal experiments in China West Normal University, and the experimental protocols complied with the current laws of China concerning animal experimentation (Approval date: 20 September 2015). A total of 100 individuals (50 females and 50 males) were caught in mid-October 2015 from Nanchong (30°49′ N, 106°03′ E, 251 metre above sea level) in Sichuan Province, China. All individuals were kept in a pond (length × width × depth: 3 m × 2 m × 1 m). After 48 h, we randomly selected 10 active females and 10 active males. Individuals were transferred to the laboratory and kept individually in a rectangular tank (0.5 m × 0.4 m × 0.4 m; Chahua, Fujian, China), and then killed by single-pithing [43,44,45,46,47]. We measured the body size (snout-vent length: SVL) of each individual to the nearest 0.01 mm with a calliper (SHANGGOMG, Shanghai, China). Body mass and fat-body mass were measured to the nearest 0.1 mg with an electronic balance (HANGPING FA2204B, Shanghai, China). In late December 2015, we randomly captured 20 individuals (10 torpid females and 10 torpid males) from the pond where all individuals had been hibernating for 40 days. We anesthetized all individuals and killed them using single-pithing [48,49,50,51,52,53]. We promptly performed surgical procedures to protect RNA from degradation. The livers from active and torpid toads were rapidly excised, flash frozen in liquid nitrogen, and then stored at −80 °C until processing for RNA isolation.

### 4.2. RNA Isolation and cDNA Library Construction

Total RNA in livers for all individuals was extracted using Trizol (Life Technologies Corporation, Carlsbad, CA, USA). RNA concentration was then measured with the use of a NanoDrop 2000 system (Thermo, Wilmington, SD, USA). We assessed RNA integrity using the RNA Nano 6000 Assay Kit of the Agilent Bioanalyzer 2100 system (Agilent Technologies, Santa Clara, CA, USA). Biomarker Technologies Corporation (Beijing, China) used an Illumina Genome Analyzer II platform (Illumina, San Diego, CA, USA) to perform the deep sequencing of Total RNA. A total amount of 1 μg RNA per sample was provided for input material for the RNA sample preparations. We used NEBNext UltraTM RNA Library Prep Kit for Illumina (NEB, USA) to generate sequencing libraries and added index codes to attribute sequences to each sample. Briefly, we used poly-T oligo-attached magnetic beads to purify mRNA from total RNA. Fragmentation was conducted using divalent cations under increased temperature in NEBNext first strand synthesis reaction buffer (5×). First strand cDNA synthesis was performed using random hexamer primer and M-MuLV reverse transcriptase. Second strand cDNA was subsequently synthesized using DNA polymerase I and RNase H. Remained overhangs were transferred to blunt ends through exonuclease/polymerase activities. We ligated the NEBNext adaptor with a hairpin loop structure to prepare for hybridization after adenylation of 3′ ends of DNA fragments. The library fragments were purified using an AMPure XP system (Beckman Coulter, Beverly, MA, USA) to choose cDNA fragments of, preferentially, 240 bp in length. We used the 3 μL USER enzyme (NEB, USA) with size-selected, adaptor-ligated cDNA at 37 °C for 15 min followed by 5 min at 95 °C before PCR. We performed the PCR with Phusion High-Fidelity DNA polymerase, Universal PCR primers and Index (X) Primer. Finally, we used an AMPure XP system to purify PCR products and assessed library quality using the Agilent Bioanalyzer 2100 system. We constructed four cDNA libraries (active females (AF), active males (AM), torpid females (TF), and torpid males(TM)) with the Super-Script^®^ Double-Stranded cDNA Synthesis Kit (Invitrogen, cat. no. 11917-010, Carlsbad, CA, USA). Raw sequence data generated by Illumina pipeline were deposited into Short Read Archive (SRA) database of NCBI with the accession no. SRR5344011 (active females), no. SRR5344010 (active males), no. SRR5344009 (torpid females), and no. SRR5344008 (torpid males).

### 4.3. Transcriptome Sequencing and Assembly

Sequencing was performed via a paired-end 125 cycle rapid run on two lanes of the Illumina HiSeq2500 (Illumina, San Diego, CA, USA), generating more than 89.59 million pairs of reads as desired. We separately assembled de novo from transcriptomes using Trinity [54]. The left files (read1 files) and right files (read2 files) from all libraries/samples were pooled into one large left.fq file and one large right.fq file, respectively. Transcriptome assembly was accomplished on the basis of both the left.fq and right.fq with min_kmer_cov set to 2 on the basis of default, and all other parameters set to default. In particular, long fragments without N (named contigs) initially consisted of clean reads with a certain overlap length. We used the TGICL software [55] to cluster related contigs in order to produce unigenes (without N) that cannot be extended on either end, and we removed redundancies to acquire non-redundant unigenes. We then separately mapped raw reads from four libraries to pre-assembled contigs (length > 500) using BWA v0.6.1-r104 [56,57], with two critical parameters: less than five mismatches and no gap. High-quality clean reads were obtained by removing the adaptor sequences, duplicated sequences, ambiguous reads (‘N’), and low-quality reads (including the removal of ‘N’ ratio greater than 10% of reads, the base number of the quality value *Q* ≤ 10 is removed by reads of more than 50% of the entire read.).

### 4.4. Gene Annotation

To retrieve protein functional annotations on the baisis of sequence similarity, we searched the unigene sequences of the four groups by BLASTX against the NCBI nonredundant (NR), Gene Ontology (GO), Kyoto Encyclopedia of Genes and Genomes (KEGG), Cluster of Orthologous Group of proteins (COG/KOG), Swiss-Prot protein (Swiss-Prot), and Protein family (PFAM) databases (*E*-value ≤ 1 × 10^−5^). We determined the direction of the unigene sequences using high-priority databases. We then decided the sequence direction of the unigenes that could not be aligned to any of the above databases using the ESTScan software [58]. We assigned GO terms to each sequence annotated using BLASTX against the NR database on the basis of the Blast2GO program with the *E*-value threshold of 1 × 10^−5^ for further functional categorization. The distribution of the GO functional classification of the unigenes was plotted by the Web Gene Ontology Annotation Plot (WEGO) software [59]. We analysed inner-cell metabolic pathways and the related gene function using BLASTX through assigning the unigenes to KEGG pathway annotations.

### 4.5. Analysis of the Functional Enrichment of DEGs

Read counts of every gene were estimated through using RNA-Seq by Expectation Maximization (RSEM) [60] which was nested in the Trinity package [54]. Then, we calculated FPKM (fragments per kilobase of exon per million fragments mapped) values to normalize the read counts so that the gene expression difference between two different samples was able to be compared. Here, we detected differentially expressed genes by using EdgeR method [61]. On the basis of applying Benjamini-Hochberg correction [62] for multiple test, FDR ≤ 0.01 and the absolute value of log_2_ fold change ≥2 were set as the thresholds for the significance of the gene expression difference between the samples.

### 4.6. Quantitative Real-Time PCR

To test the validity of our measurements, qRT-PCR was performed to detect the relative messenger RNA (mRNA) expression levels of 18 randomly selected genes which were down-regulated (c106287.graph_c0, c111514.graph_c0) or up-regulated (c100636. graph_c0, c102204.graph_c0) between active females and active males (AF-vs.-AM) in the transcriptome sequencing, down-regulated (c80857.graph_c0) or up-regulated (c122853.graph_c0, c122834.graph_c0) between active females and torpid females (AF-vs.-TF), down-regulated (c85945.graph_c0, c76918.graph_c0) or up-regulated (c114573.graph_c0, c109992.graph_c1, c20664.graph_c0) between active males and torpid males (AM-vs.-TM), down-regulated (c122942.graph_c0, c77297.graph_c0, c101480. graph_c0) or up-regulated (c111770.graph_c0, c102232.graph_c1, c123340.graph_c0) between torpid females and torpid males (TF-vs.-TM). The α-actin gene was selected as the house-keeping gene [63]. The primer pairs for the 18 genes and house-keeping α-actin gene for *B. gargarizans* are listed in Table S5, including their sequences, and product lengths. Messenger RNA samples from the livers of 40 individuals (10 active females, 10 active males, 10 torpid females, 10 torpid males), were converted to cDNA templates. qRT-PCRs were performed using a StepOne Real-Time PCR System (Applied Biosystems, Thermo Fisher Scientific, Shanghai, China) and an automatic threshold calculated by the StepOne software v2.1 (Applied Biosystems, Thermo Fisher Scientific, Shanghai, China). For each sample, three technical replicates of each PCR reaction were run. For each target gene, reactions of all biological replicates (i.e., all samples) in the active and torpid state (or female and male) were completed on one plate to eliminate inter-run heterogenity. Each 20 μL PCR mixture reaction contained 10 μL SybrGreen qPCR Master Mix, 7.2 μL ddH_2_O, 2 μL cDNA template and 0.4 μL of each primer. PCRs were carried out using the following parameters: 95 °C × 3 min, 45 cycles of 95 °C × 7 s, 57 °C × 10 s, 72 °C × 15 s. The standard deviation between two reactions of each sample was less than 0.5, which means the CT (crossing threshold) value of each sample was used in further analyses. The relative quantity was calculated by using 2^−ΔΔ*C*T^ method [64].

## 5. Conclusions

To sum up, we used the Illumina HiSeq 2500 platform to sequence liver transcriptomes of active and torpid *B. gargarizans* to gain insights into changes in gene expression patterns in hibernating female and male toads. We discovered that 395 genes, mainly involved in stress responses, including contractile proteins, were significantly up-regulated, whereas 1004 genes mainly involved in metabolic depression and shifts in energy utilization were down-regulated in torpid females. Similar patterns were recoded in comparison of torpid and active males but, in general, fewer genes were markedly expressed in males than females. More specifically, 695 genes were differentially expressed between active females and active males: of these expression of 655 genes were significantly down-regulated in activemales. Only 374 differentially expressed genes were identified between torpid females and males, but again, most of these (252 genes) were significantly down-regulated in torpid males. The genes encoding an important enzyme (NADPH-cytochrome P450 reductase isoform X1) were down-regulated so as to achieve the accumulation of hepatic lipids, thus, the metabolic rate would be reduced and the survival of poikilotherms living in tough winter conditions would be enhanced. However, the metabolic rate of females was higher than that of males as they were carrying developing eggs during the hibernating period. Moreover, the up-regulation of genes encoding contractile proteins might be caused by the increasing demands of oxygen for torpid toads during hibernation. Further, many *HSP* were up-regulated in torpor (cf. comparisons: AF-vs.-TF and AM-vs.-TM), indicating that *HSP* may be responsible for maintaining tissue-specific functions via the protein homeostasis pathway during torpor. Totally, the findings suggest that levels and patterns of gene expression in liver of anurans are significantly influenced by torpor, and that there are significant differences in gene expression between males and females.

## Figures and Tables

**Table 1:** Sequencing, assembly, and mapping statistics for samples of active and torpid females and males of Asiatic toads. Mapped ratio refers to percentage of mapped reads in clean reads.

	Active Females	Active Males		Torpid Females	Torpid Males
Sequencing					
Base number	5,240,784,114	6,460,736,308		5,426,895,884	5,393,246,014
Clean read number	20,836,817	25,678,062		21,610,351	21,463,798
GC content (%)	48.33	47.42		47.47	47.60
Assembly					
Contigs (>500 bp)			33,976		
Transcript (>500 bp)			46,560		
Unigene (>500 bp)			27,349		
Mapping					
Mapped ratio (%)	78.22	78.53		80.28	78.65

**Table 2:** The genes with the greatest fold change between active females and males.

Unigene ID	Nr Annotation	RPKM (Females)	RPKM (Males)	log_2_FC
Down-regulated genes				
c120255.graph_c0	Hydroxyacid oxidase 1	40.157	0	−10.029
c114360.graph_c0	Aldolase A	31.087	0	−9.920
c107620.graph_c0	Photosystem II 10 kDa polypeptide, chloroplast	68.956	0	−9.676
c119772.graph_c0	Glyceraldehyde-3-phosphate dehydrogenase-like	17.887	0	−9.281
c110474.graph_c0	Glycine-rich RNA-binding protein GRP1A-like, partial	50.161	0	−9.146
c120235.graph_c0	Nicotinamide adenine dinucleotide phosphate (NADPH)-cytochrome P450 reductase isoform X1	21.024	0	−8.941
c120129.graph_c0	Chaperone protein ClpB-like, partial	10.981	0	−8.913
c112955.graph_c0	Thioredoxin-like isoform X2	16.395	0	−8.745
c20631.graph_c0	Uncharacterized protein LOC102345338	17.348	0	−8.651
c118905.graph_c0	Alanine aminotransferase 2-like isoform X3	10.463	0	−8.560
Up-regulated genes				
c75071.graph_c0	Uncharacterized protein LOC100127559	0.115	24.499	6.773
c111830.graph_c0	MOSC domain-containing protein 1, mitochondrial-like	0	3.386	6.176
c107611.graph_c0	Keratin	0.070	3.953	5.201
c96812.graph_c0	MGC81526 protein	0.262	11.755	5.006
c123079.graph_c0	Actin-85C-like, partial	0.465	9.786	3.484
c123016.graph_c0	Fast troponin I	0.279	2.091	2.529
c109275.graph_c0	Novel protein similar to human angiopoietin-like ANGPTL	2.538	15.979	2.456
c120261.graph_c0	Choline transporter-like protein 4	0.415	2.479	2.304
c119575.graph_c1	SLC12A8 cation-chloride cotransporter	2.349	13.111	2.295
c100092.graph_c0	Hemoglobin A chain	2.404	13.614	2.287

**Table 3:** The genes with the greatest fold change between active and torpid females.

Unigene ID	Nr Annotation	RPKM (Active)	RPKM (Torpid)	log_2_FC
Down-regulated genes				
c120255.graph_c0	Hydroxyacid oxidase 1	40.157	0	−9.328
c114360.graph_c0	Aldolase A	31.087	0	−9.219
c107620.graph_c0	Photosystem II 10 kDa polypeptide, chloroplast	68.956	0	−8.975
c119772.graph_c0	Glyceraldehyde-3-phosphate dehydrogenase-like	17.887	0	−8.580
c110474.graph_c0	Glycine-rich RNA-binding protein GRP1A-like, partial	50.161	0	−8.445
c120235.graph_c0	NADPH-cytochrome P450 reductase isoform X1	21.024	0	−8.241
c120129.graph_c0	Chaperone protein ClpB-like, partial	10.981	0	−8.213
c112955.graph_c0	Thioredoxin-like isoform X2	16.395	0	−8.045
c121279.graph_c0	Transketolase-like	7.695	0	−7.813
c118687.graph_c0	Serine hydroxymethyltransferase, mitochondrial isoform X1	10.790	0	−7.792
Up-regulated genes				
c87275.graph_c0	ATPase, Ca++ transporting, cardiac muscle, fast twitch 1	0.016	182.924	12.085
c115995.graph_c0	Myosin heavy chain IIa	0.020	28.514	9.528
c122569.graph_c1	Titin isoform X4	0	5.402	9.170
c87310.graph_c0	Troponin T	0.048	37.017	8.226
c122549.graph_c0	Obscurin-like	0.024	16.880	8.059
c95821.graph_c0	Telethonin isoformX2	0	21.439	7.848
c26408.graph_c0	Myosin light chain, phosphorylatable, fast skeletal muscle	0	27.037	7.814
c122942.graph_c0	Cysteine and glycine-rich protein 3	0	25.036	7.756
c74187.graph_c0	Uncharacterized protein LOC100036938	0	8.947	7.638
c105949.graph_c0	Myozenin-1-like	0	10.209	7.449

**Table 4:** The genes with the greatest fold change between active and torpid males.

Unigene ID	Nr Annotation	RPKM (Active)	RPKM (Torpid)	log_2_FC
Down-regulated genes				
c107611.graph_c0	Keratin	3.953	0	−6.375
c75071.graph_c0	Uncharacterized protein LOC100127559	24.499	0.223	−6.108
c81220.graph_c0	Heat shock protein HSP 90-beta	4.027	0	−5.709
c85444.graph_c0	Tyrosine aminotransferase	2.588	0	−5.518
c85945.graph_c0	Tubulin alpha-1A chain-like	3.081	0	−5.162
c111752.graph_c0	Long-chain-fatty-acid--CoA ligase ACSBG1 isoform X1	12.757	0.365	−5.114
c115272.graph_c0	L-threonine 3-dehydrogenase, mitochondrial-like	1.904	0	−4.874
c70612.graph_c0	Uncharacterized protein LOC101731022	4.881	0.140	−4.810
c80857.graph_c0	Uncharacterized protein LOC102222694	1.731	0	−4.688
c26374.graph_c0	Heat shock 70 kDa protein 1	1.931	0	−4.475
Up-regulated genes				
c20631.graph_c0	Uncharacterized protein LOC102345338	0	26.152	8.184
c122917.graph_c0	Full = Olfactory protein; Flags: Precursor	0.165	62.383	7.752
c114573.graph_c0	Mucin-5AC-like	0	7.871	6.562
c122235.graph_c0	Uncharacterized protein LOC101734130, partial	0.106	10.236	5.549
c26943.graph_c0	Stc2 protein	0.476	29.446	5.542
c119368.graph_c0	Zinc finger BED domain-containing protein 1-like isoform X1	0.204	8.588	5.026
c99486.graph_c0	Serine/threonine-protein kinase DCLK3	0.023	1.511	4.438
c107619.graph_c0	DnaJ (Hsp40) homolog, subfamily A, member 4, gene 1	0.152	4.417	4.212
c106739.graph_c0	Sperm-associated antigen 17	0.164	5.172	4.199
c122333.graph_c0	Heat shock protein 70	0.513	11.259	4.180

**Table 5:** The genes with the greatest fold change between torpid females and males.

Unigene ID	Nr Annotation	RPKM (Females)	RPKM (Males)	log_2_FC
Down-regulated genes				
c87275.graph_c0	ATPase, Ca++ transporting, cardiac muscle, fast twitch 1	182.924	0.207	−9.778
c121033.graph_c0	Nebulin-related-anchoring protein	20.819	0	−9.610
c59975.graph_c0	Cold shock domain protein A, partial	39.894	0.040	−8.993
c95821.graph_c0	Telethonin isoformX2	21.439	0	−8.384
c122942.graph_c0	Cysteine and glycine-rich protein 3 (cardiac LIM protein)	25.036	0	−8.291
c74187.graph_c0	Uncharacterized protein LOC100036938	8.947	0	−8.173
c122853.graph_c0	Ribosomal protein L3-like	39.071	0.096	−8.159
c115995.graph_c0	Myosin heavy chain IIa	28.514	0.101	−8.108
c105949.graph_c0	Myozenin-1-like	10.209	0	−7.984
c101328.graph_c0	Tripartite motif containing 54	11.995	0	−7.921
Up-regulated genes				
c122917.graph_c0	Full = 0l factory protein; Flags: Precursor	0.064	62.383	8.801
c111770.graph_c0	Uncharacterized protein LOC101732866	0	13.864	8.469
c96835.graph_c0	Phosphoenolpyruvate carboxykinase, cytosolic	0.228	8.825	5.009
c123029.graph_c0	Carboxypeptidase B1 (tissue) precursor	1.060	31.190	4.709
c26943.graph_c0	Stc2 protein	0.987	29.446	4.701
c19061.graph_c0	Growth factor receptor-bound protein 14	0	3.031	4.434
c102557.graph_c0	Fatty acyl-CoA hydrolase precursor, medium chain-like	0.246	10.881	4.379
c89882.graph_c0	Homeobox protein SIX6-like	0.152	6.725	4.379
c106739.graph_c0	Sperm-associated antigen 17	0.191	5.172	4.245
c125726.graph_c0	LOW QUALITY PROTEIN: HEAT repeat-containing protein 4	0	1.640	4.233

**Table 6:** Synopsis of functional annotation of unigenes of the *Bufo gargarizans* transcriptome. AF, AM, TF, and TM refer to active females, active males, torpid females, and torpid males, respectively.

Databases	All Annotated Transcripts	300–1000 (bp)	>1000 (bp)	AF-vs.-AM Annotated Transcripts	AF-vs.-TF Annotated Transcripts	AM-vs.-TM Annotated Transcripts	TF-vs.-TM Annotated Transcripts
COG	5760	1610	3686	237	355	104	46
GO	10,554	3154	6735	115	329	152	117
KEGG	10,798	3390	6551	149	380	157	136
KOG	13,424	4281	7939	309	565	191	138
PFAM	15,330	4818	9370	455	783	266	190
Swiss-Prot	10,902	3263	6950	167	421	183	163
NR	20,046	7283	10,699	272	666	335	234
All_Annotated	21,261	7940	10,787	502	903	342	234

**Table 7:** Genes in significant Gene Ontology categories of the biological processes.

GO Category	NR Annotation	Unigene ID	AF-vs-AM	AF-vs-TF	AM-vs-TM	TF-vs-TM
			log_2_FC	log_2_FC	log_2_FC	log_2_FC
Carbohydrate metabolic process (GO:0005975)	Malate dehydrogenase, mitochondrial isoform X1	c117841	−8.099	−7.401		
Glycolytic process (GO:0006096)	Glyceraldehyde-3-phosphate dehydrogenase-like	c119772	−9.282	−8.581		
	Glyceraldehyde 3-phosphate dehydrogenase, partial	c73032	−5.337	−4.657		
	Glyceraldehyde-3-phosphate dehydrogenase	c102216	−4.671	−4.004		
	Phosphoglycerate kinase 1	c108799	−5.861	−5.174		
	Fructose-bisphosphate aldolase A	c117125	−8.378	−7.679		
Cellular amino acid metabolic process (GO:0006520)	Glycine dehydrogenase [decarboxylating], mitochondrial	c109713	−7.590	−6.893		
	Dopa decarboxylase (aromatic L-amino acid decarboxylase)	c21604		−3.845		
	Tyrosine aminotransferase	c85444		−5.009	−5.519	
Very long-chain fatty acid metabolic process (GO: 0000038)	Long-chain-fatty-acid-CoA ligase ACSBG1 isoform X1	c111752		−4.667	−5.114	
Fatty acid metabolic process (GO: 0006631)	Acyl-CoA synthetase family member 2, mitochondrial	c109605		−2.281		
Positive regulation of fast-twitch skeletal muscle fiber contraction (GO: 0031448)	ATPase, Ca^++^ transporting, cardiac muscle, fast twitch 1	c87275		12.086		−9.778
Response to stress (GO: 0006950)	Heat shock protein 90kDa alpha (cytosolic), class A member 1, gene 2	c88789		3.994		
	Cardiovascular heat shock protein	c112677		3.869		
	Heat shock protein 70	c122333		2.441	4.181	
	Heat shock 22kDa protein 8	c107729		5.073		
	Heat shock protein 90 alpha	c107796			2.023	
Response to heat (GO:0009408)	Heat shock protein beta-1	c51360		6.034		

## Supplementary Materials

Supplementary Materials can be found at http://www.mdpi.com/1422-0067/19/8/2363/s1.

## Abbreviations

AF	Active females
AM	Active males
TF	Torpid females
TM	Torpid males
FPKM	Fragments per kilobase of exon per million fragments mapped
BMR	Basal metabolic rate
FDR	False discovery rate
RPKM	Reads Per Kilobase per Million mapped reads
WEGO	Web Gene Ontology Annotation Plot
RSEM	RNA-Seq by Expectation Maximization
CT	Crossing threshold
NGS	Next-generation sequencing
cDNA	Complementary DNA
NADPH	Nicotinamide adenine dinucleotide phosphate
HSP	Heat shock protein
NR	NCBI nonredundant
KEGG	Kyoto Encyclopedia of Genes and Genomes
GO	Gene Ontology
COG	Cluster of Orthologous Group of proteins
