# Supplementary material for: Modulation of Gene Expression in Liver of Hibernating Asiatic Toads (Bufo gargarizans)
Source: Int J Mol Sci. 2018 Aug 10;19(8):2363. doi: 10.3390/ijms19082363 (PMC6121651; doi:10.3390/ijms19082363)
Supplement: Supplementary file 1 [file ijms-19-02363-s001.zip › ╓╨╗¬≤╕≥▄╫¬┬╝╫Θ╬─╒┬╨▐╕─░μ/Supplementary Materials.docx]

**Supplementary Materials：**

**Table S1** List of differentially expressed genes identified in this study.

**Table S2** The common differentially expressed genes between active female vs torpid female and active male vs torpid male.

**Table S3-4** Descriptive information about the means and standard deviations of body mass and fat-body mass of Asiatic toads (*Bufo gargarizans*).

**Table S5** The primers designed for qRT-PCR.

**Figure S1** Distribution of assembly contig lengths.

**Figure S2** GO enrichment analysis of differentially expressed genes in Asiatic toad liver in different groups.
