# Supplementary material for: Modulation of Gene Expression in Liver of Hibernating Asiatic Toads (Bufo gargarizans)
Source: Int J Mol Sci. 2018 Aug 10;19(8):2363. doi: 10.3390/ijms19082363 (PMC6121651; doi:10.3390/ijms19082363)
Supplement: Supplementary file 1 [file ijms-19-02363-s001.zip › ╓╨╗¬≤╕≥▄╫¬┬╝╫Θ╬─╒┬╨▐╕─░μ/Table S3ú1⁄4S4.pdf]

Table S3. Descriptive information about the means and standard deviations of body mass and fat-body mass of Asiatic toads (*Bufo gargarizans*).

	Active females (n=10)	Active males (n=10)	Torpid females (n=10)	Torpid males (n=10)
Body mass (g)	207.84±41.49	161.73±27.64	252.33±23.48	158.96±19.90
Fat-body mass (g)	0.0785±0.0343	0.1017±0.0637	0.0754±0.0219	0.0468±0.0310

Table S4. We used the two way ANOVA to test difference and found that significant difference in body mass between males and females and active and torpid. However, there was significant difference only in fat-body mass between active and torpid, but not difference between males and females. When controlling the body mass, there is significant difference only in fat-body mass between active and torpid, but not difference between males and females.

	Females-vs-Males		Active-vs-Torpid		Interaction of sex and state	
	F	P	F	P	F	P
Body mass	56.665	<0.001	5.069	0.031	6.504	0.015
Fat-body mass	0.045	0.834	5.051	0.031	4.002	0.053
Relative fat-body mass	0.923	0.343	6.882	0.013	1.738	0.196
