# Supplementary material for: Modulation of Gene Expression in Liver of Hibernating Asiatic Toads (Bufo gargarizans)
Source: Int J Mol Sci. 2018 Aug 10;19(8):2363. doi: 10.3390/ijms19082363 (PMC6121651; doi:10.3390/ijms19082363)
Supplement: Supplementary file 1 [file ijms-19-02363-s001.zip › ╓╨╗¬≤╕≥▄╫¬┬╝╫Θ╬─╒┬╨▐╕─░μ/Table S5.pdf]

Table S5. The primers designed for qRT-PCR.

Gene	Primer	Base sequence (5’- 3’)	Products (bp)
α-actin	Sense	GCTATGAACTGCCTGATGGTC	133
	Anti-sense	TGATGCTGTTGTAGGTGGTCT	
c106287.graph_c0	Sense	CGCTGAGGAACGATTTACG	139
	Anti-sense	GCGGTTGATGCCTATGTGA	
c111514.graph_c0	Sense	CTACGACCCTCTTGTTACCCTC	197
	Anti-sense	CTTTCGCATCCCACTGTATTTA	
c100636.graph_c0	Sense	ATCCAGCAATGACTACATACCG	172
	Anti-sense	ACCCATAAATCCGCACCAG	
c102204.graph_c0	Sense	AGGTGCTGTGCTTTATTTCGT	178
	Anti-sense	GATTTGCTCTGTTTGATTATGGAC	
C85945.graph_c0	Sense	GGTCGCACTTGGTCATCATC	137
	Anti-sense	GTACCCGCGTATCCACTTTG	
c122853.graph_c0	Sense	CCCATAAGCGAAGCAAACG	90
	Anti-sense	CGAGGAAGGCTGTAAGGTGAA	
c122834.graph_c0	Sense	GTGCTATGTCGCTCTTGACTTTG	156
	Anti-sense	CCAATAAATGATGGCTGGAAGA	
c80857.graph_c0	Sense	TGCGGAAGCAAATGTCGTA	146
	Anti-sense	GACCTTCTCCATCATCCCGT	
c114573.graph_c0	Sense	TGGCTGCTGCGAGAAATAAA	202
	Anti-sense	TTGATGGCGTGGGTTTGTC	
c109992.graph_c1	Sense	GGTCCCGCTGAATGAAGAT	99
	Anti-sense	GATTGACTCACCTCGTCCACT	
c20664.graph_c0	Sense	CCCAGTGAGATAGGGCAGAA	187
	Anti-sense	GGAACAACGAACCAGACACAT	
c76918.graph_c0	Sense	GACAAGACCCCTTTTCTCGAT	110
	Anti-sense	GTTGTCCCACCATAGCAAAGTA	

c111770.graph_c0	Sense	AAAGCAATCCACTCCCTCAT	183
	Anti-sense	AATCGGTCTCATAGTGGGTTG	
c122942.graph_c0	Sense	TTTCTCCCATAACAGGACTTGC	151
	Anti-sense	AGACGGTTTACCACGCAGAG	
c102232.graph_c1	Sense	CCCCAGGCAGATTTCCATA	180
	Anti-sense	AAGGTATCCTCTATCCCACAGC	
c77297.graph_c0	Sense	CGCAGTTTCTAAAGCAAGCC	162
	Anti-sense	GGAGCGGAGCATACCCATA	
c123340.graph_c0	Sense	GCAAATCAGACGACAAATCATC	164
	Anti-sense	CACTGGTGGTGAGTTGGAAAT	
c101480.graph_c0	Sense	GATGCCAACCGCCATAGAA	229
	Anti-sense	CCAAGCTGTAAAGCCACGATA	

Primer pairs of α-actin was obtained in Wang et al. 2015.
