# Supplementary figures and images for: Modulation of Gene Expression in Liver of Hibernating Asiatic Toads (Bufo gargarizans)
Source: Int J Mol Sci. 2018 Aug 10;19(8):2363. doi: 10.3390/ijms19082363 (PMC6121651; doi:10.3390/ijms19082363)

# All\_Combination.contigs Length Distribution

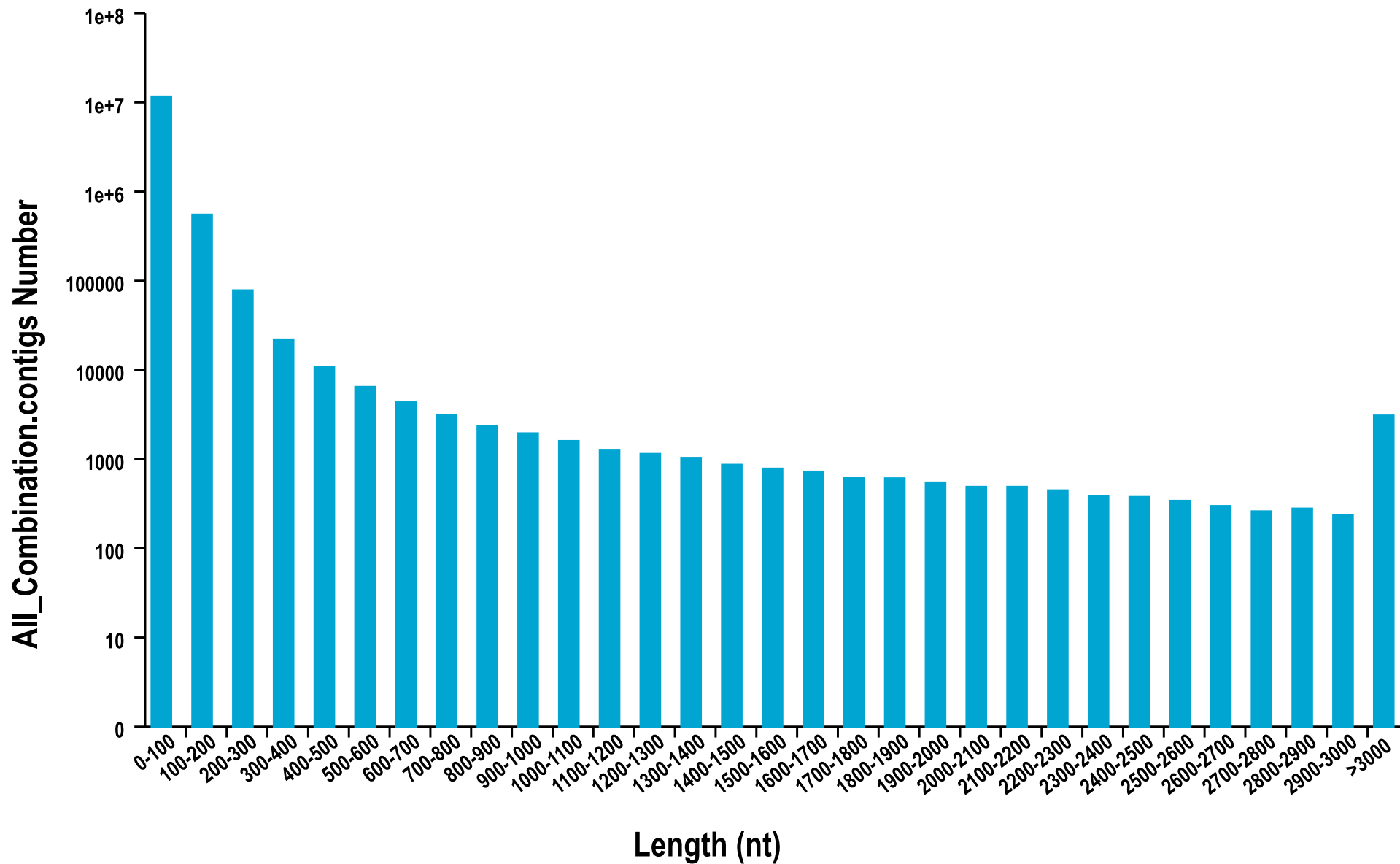

Supplement: Supplementary file 1 [file ijms-19-02363-s001.zip › ╓╨╗¬≤╕≥▄╫¬┬╝╫Θ╬─╒┬╨▐╕─░μ/Figure S1. All_Combination.contigs.distribution.pdf]

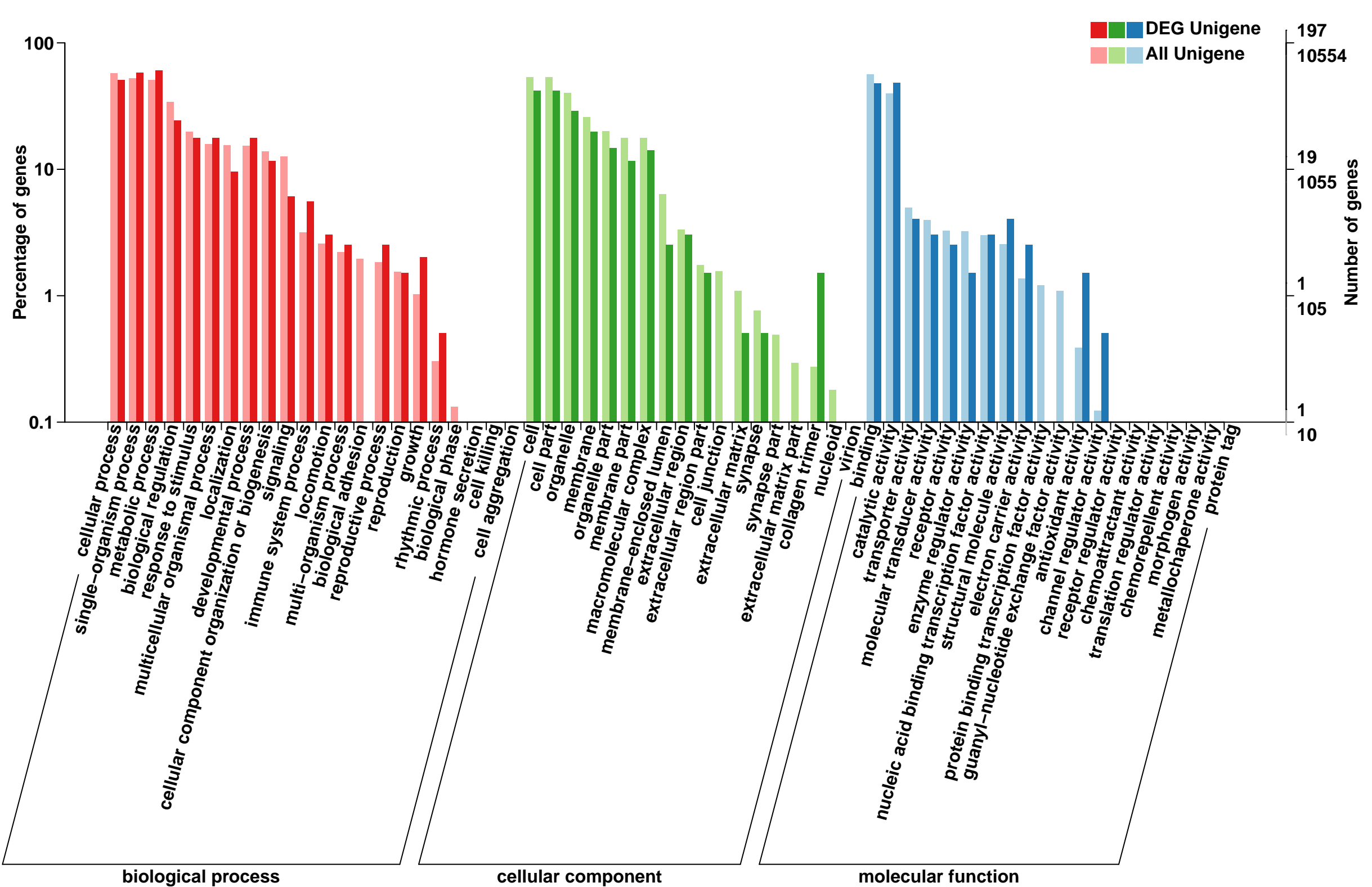

Supplement: Supplementary file 1 [file ijms-19-02363-s001.zip › ╓╨╗¬≤╕≥▄╫¬┬╝╫Θ╬─╒┬╨▐╕─░μ/Figure S2a AF_vs_AM.GO.pdf]

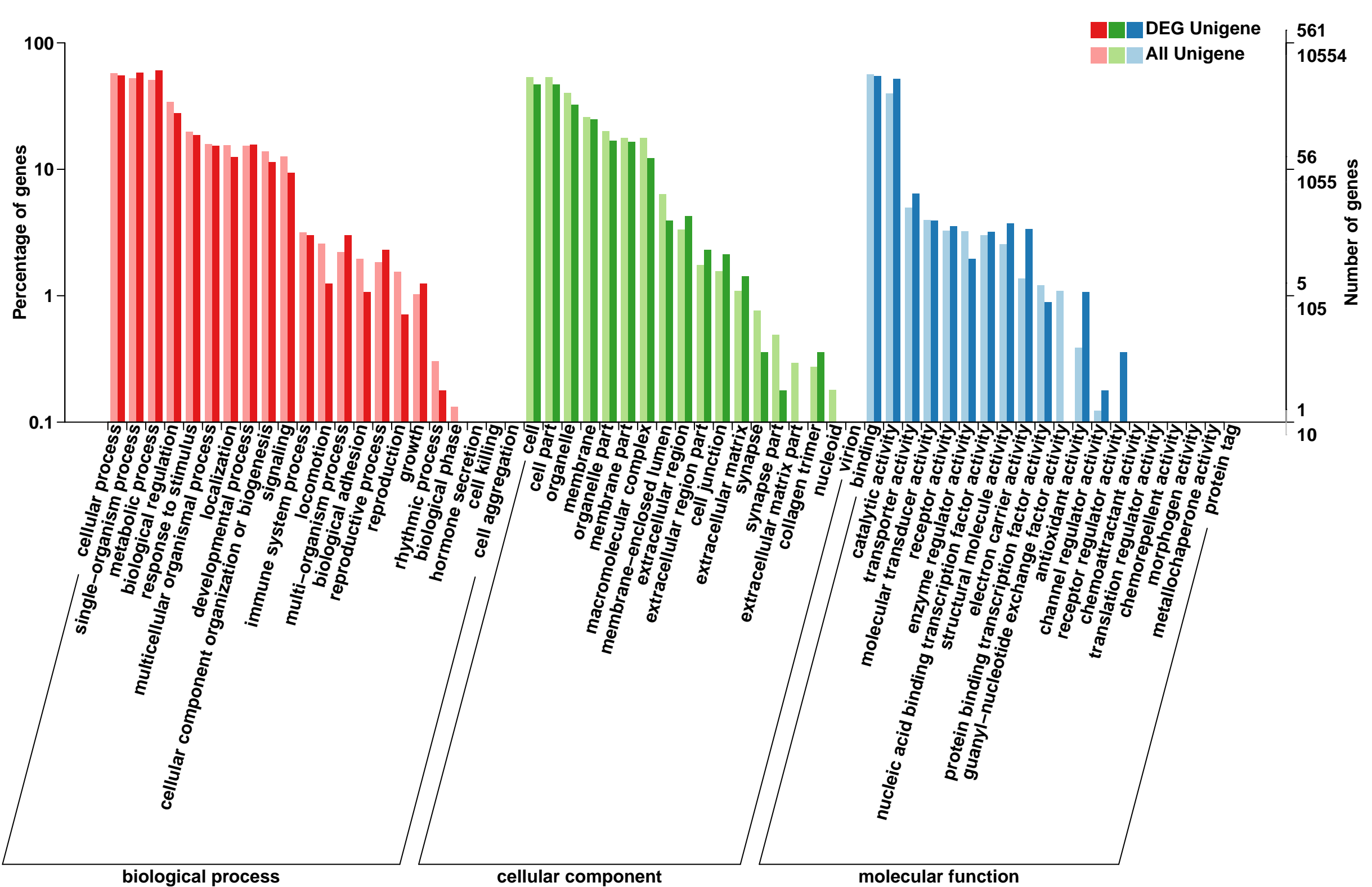

Supplement: Supplementary file 1 [file ijms-19-02363-s001.zip › ╓╨╗¬≤╕≥▄╫¬┬╝╫Θ╬─╒┬╨▐╕─░μ/Figure S2b AF_vs_TF.GO.pdf]

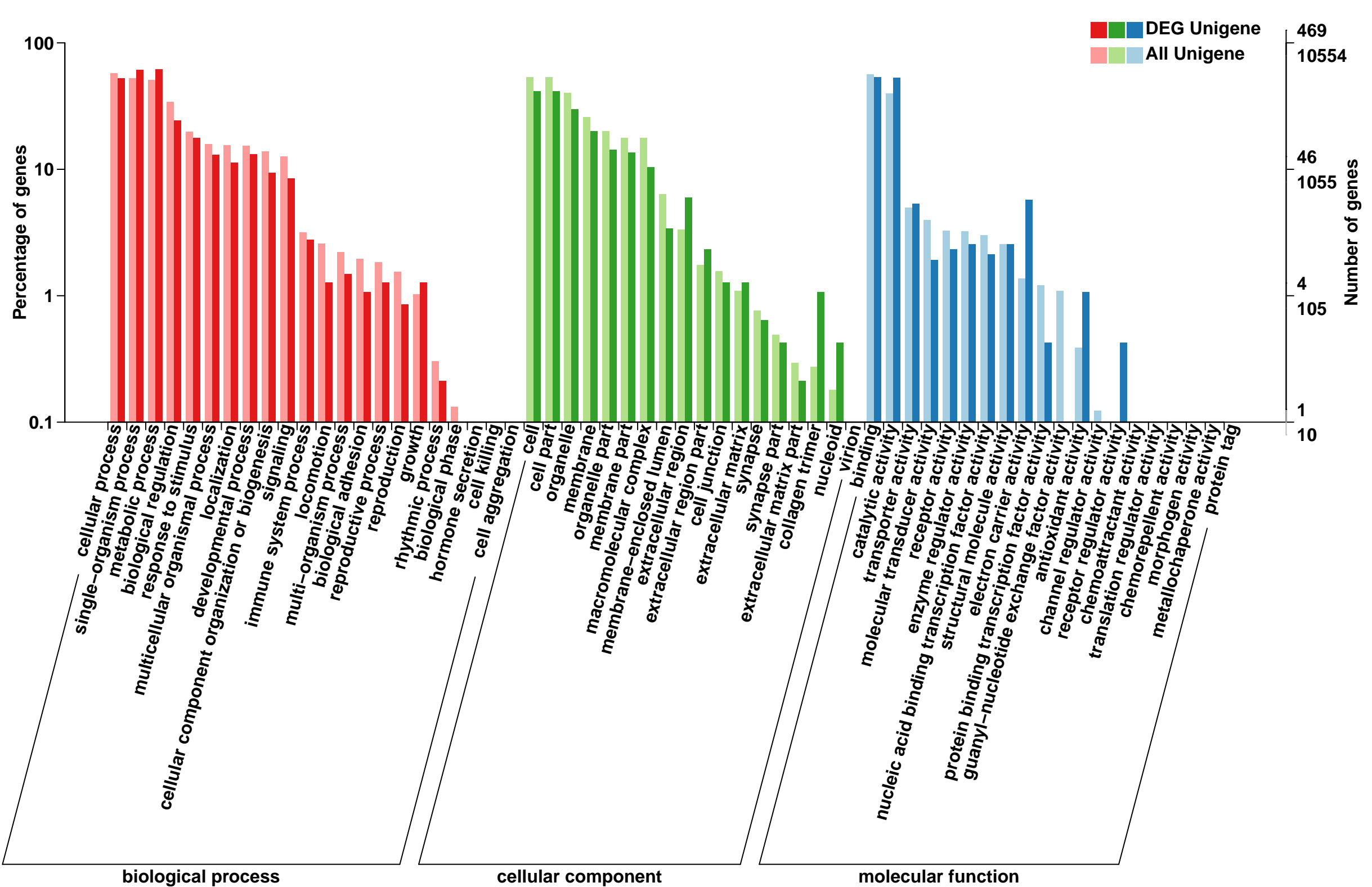

Supplement: Supplementary file 1 [file ijms-19-02363-s001.zip › ╓╨╗¬≤╕≥▄╫¬┬╝╫Θ╬─╒┬╨▐╕─░μ/Figure S2c AM_vs_TM.GO.pdf]

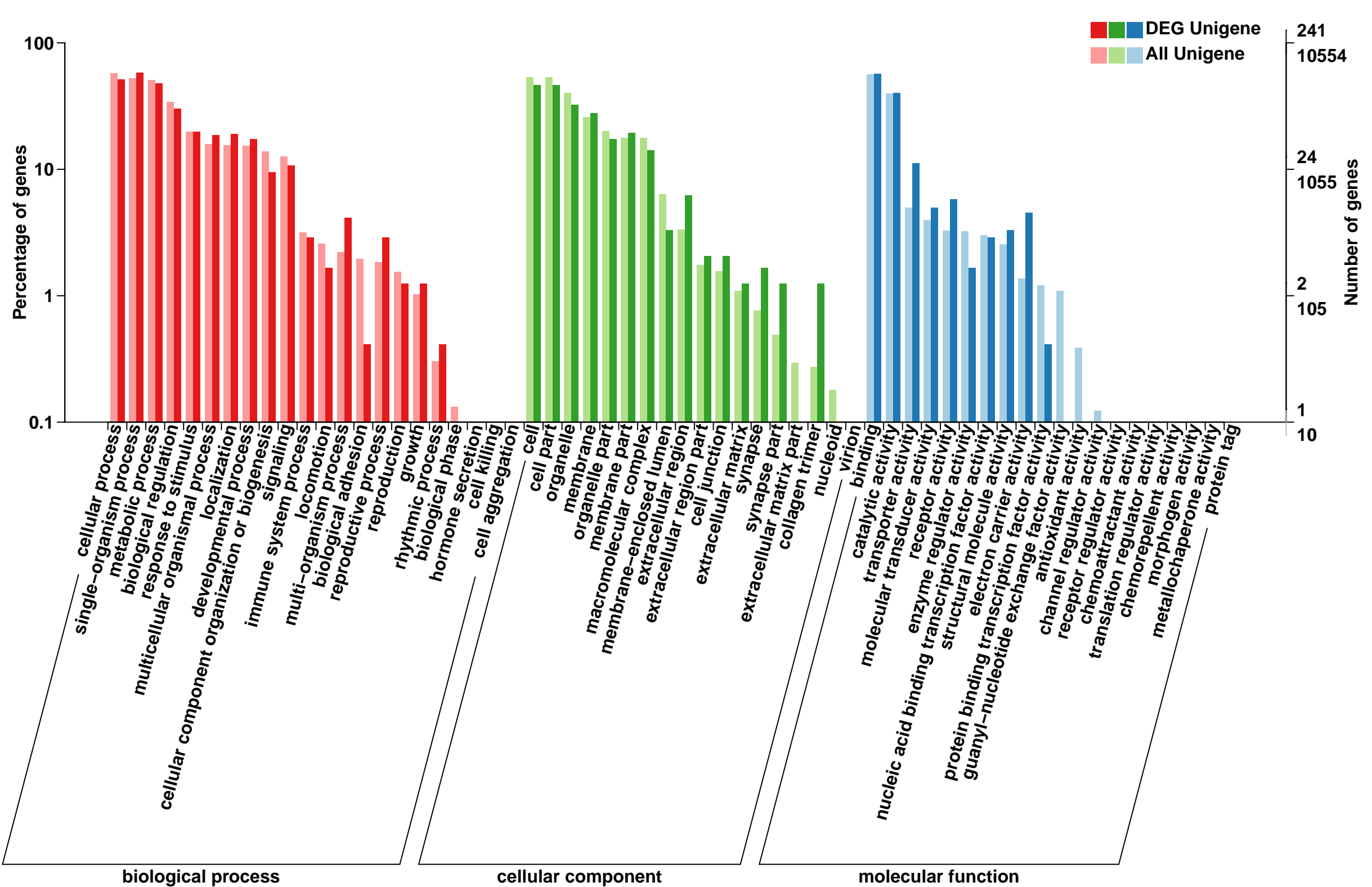

Supplement: Supplementary file 1 [file ijms-19-02363-s001.zip › ╓╨╗¬≤╕≥▄╫¬┬╝╫Θ╬─╒┬╨▐╕─░μ/Figure S2d TF_vs_TM.GO.pdf]
